# Artificial intelligence for DR screening

**Published:** 2023-07-07

**Authors:** Charles Cleland

**Affiliations:** 1 Clinical Research Fellow: International Centre for Eye Health, London School of Hygiene & Tropical Medicine, London, UK.

**AI is already being used in some diabetic retinopathy screening progammes. How does it work, and how will it affect the role of DR graders?**

Artificial intelligence software can be used to grade diabetic retinopathy (DR) images. There are several commercially available AI tools for DR screening that have been proven to be just as accurate as human graders in detecting DR.^1^

In practice, a retinal image taken with a camera is submitted to the AI software. The software can be an integrated part of the camera itself, or it can function as a separate application on a computer or laptop which is connected to the camera. The AI automatically grades the image and gives a result, typically in 30 seconds or less. The majority of AI systems are based in the ‘cloud’ (on geographically remote servers), so they must be connected to the internet to produce a report.

**“It is very unlikely that AI will completely replace the role of DR graders in the near future”**

Most AI systems give a simple response, such as “refer” or “don’t refer”, or they tell the user that the image is too low in quality and therefore ungradable. Some systems give a more detailed grade for DR, usually according to the International Clinical Diabetic Retinopathy (ICDR) severity scale.

**Figure:**
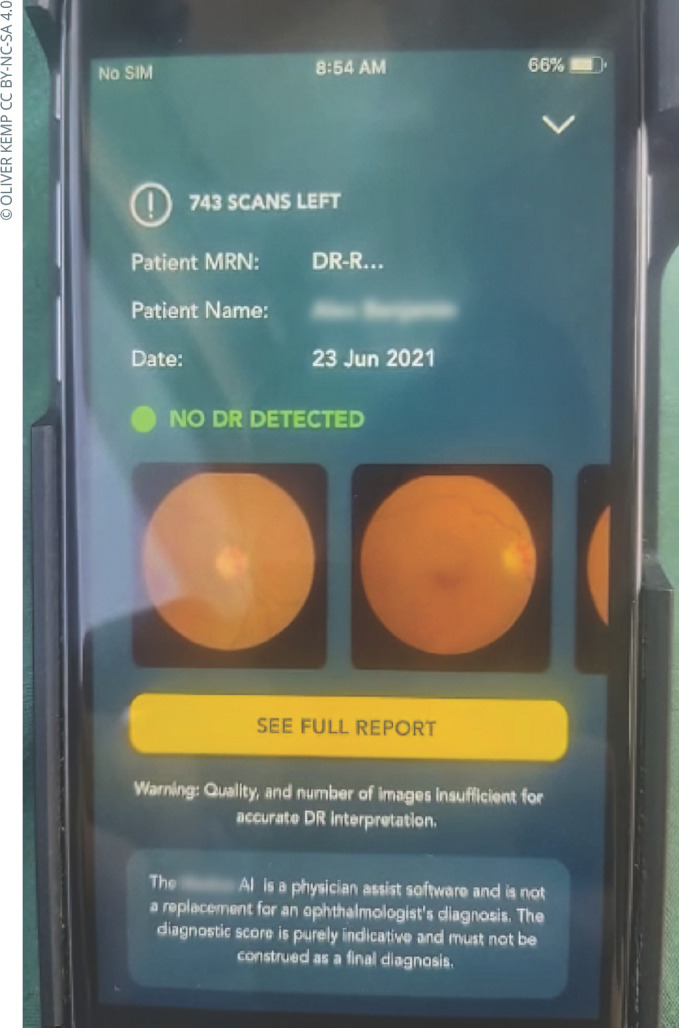
An example of a smartphone-based DR algorithm.

**Figure:**
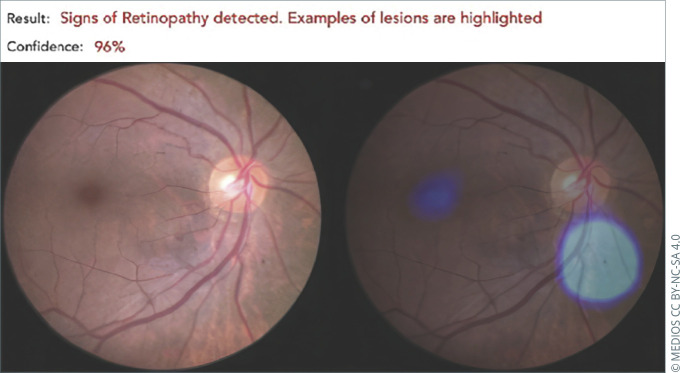
An example of the output from an AI algorithm, showing the image and a diagnosis of diabetic retinopathy (ungraded).

It is very unlikely that AI will completely replace the role of DR graders in the near future. In part, this is due to the cost of cameras, software, and other technologies, as well as ethical and legal issues.^2^^,^^3^ In addition, human graders are also needed to provide quality assurance for a programme involving AI. Patients enrolled in a screening programme need to be counselled at the point of screening by an appropriately trained staff member whose role it is to explain the results and the next steps to take.

Overall, AI is likely to become a tool that will assist DR programmes and graders to deliver effective and patient-centred DR programmes, rather than becoming a replacement for graders.

**A hybrid approach**

Some systematic DR programmes have already integrated AI into the clinical pathway. In Scotland's DR programme, for example, AI is not used to grade DR. Instead, it is used to identify **normal** retinal images, so that only those images with some DR are sent for grading by a human grader.

By first identifying those images that are entirely free of disease, and eliminating them from the workstream, AI helps to reduce the number of images that need to be reviewed by human DR graders. This enhances efficiency in the screening and grading process, without removing the need for human graders.

## References

[1] Grzybowski A Brona P Lim G. Artificial intelligence for diabetic retinopathy screening: a review. Eye. 2020; 34(3): 451–60. 31488886 10.1038/s41433-019-0566-0 PMC7055592

[2] Beede E Baylor E Hersch F. A Human-Centered Evaluation of a Deep Learning System Deployed in Clinics for the Detection of Diabetic Retinopathy. Proceedings of the 2020 CHI Conference on Human Factors in Computing Systems. Honolulu, HI, USA: Association for Computing Machinery; 2020:1–12.

[3] Mathenge WC. Artificial intelligence for diabetic retinopathy screening in Africa. Lancet Digit Health. 2019;1(1):e6-e7. 33323241 10.1016/S2589-7500(19)30009-3

